# Volunteer unrelated donor cell‐derived acute myeloid leukemia with *RUNX1‐RUNX1T1*

**DOI:** 10.1002/jha2.169

**Published:** 2021-02-09

**Authors:** Shinya Hagiwara, Shigeru Kusumoto, Asahi Ito, Ayako Masaki, Kazuhide Shiraga, Takuto Tachita, Kentaro Hirade, Kana Oiwa, Tomotaka Suzuki, Shiori Kinoshita, Masaki Ri, Yasuhiko Ito, Hirokazu Komatsu, Hiroshi Inagaki, Shinsuke Iida

**Affiliations:** ^1^ Department of Hematology and Oncology Nagoya City University Graduate School of Medical Sciences Nagoya Japan; ^2^ Department of Pathology and Molecular Diagnostics Nagoya City University Graduate School of Medical Sciences Nagoya Japan; ^3^ Department of Gastroenterology and Hematology Hirosaki University Graduate School of Medicine Hirosaki Japan; ^4^ Department of Pediatrics and Neonatology Nagoya City University Graduate School of Medical Sciences Nagoya Japan; ^5^ Nagoya City West Medical Center Pediatrics Nagoya Japan

**Keywords:** allogeneic transplantation, donor cell‐derived leukemia, volunteer unrelated donor

## Abstract

A 15‐year‐old male was diagnosed with acute myeloid leukemia with t(6;9)(p23;q34), a chimeric *DEK*‐*NUP214* fusion gene. He underwent allogeneic bone marrow transplantation (allo‐BMT) from an unrelated volunteer donor at first molecular remission. Approximately 5 years after allo‐BMT, multiple bone marrow aspirations showed increased blasts to 63%, which were positive for myeloperoxidase, CD13, CD33, CD56, and CD34. Surprisingly, t(8;21)(q22;q22.1), a chimeric *RUNX1‐RUNX1T1* (not *DEK‐NUP214)* fusion gene, was detected with full donor chimerism. To our best knowledge, this is the first case of a volunteer unrelated donor cell‐derived acute myeloid leukemia harboring a chimeric *RUNX1‐RUNX1T1* fusion gene.

**Abbreviations**
allo‐BMT: allogeneic bone marrow transplantation
AML: acute myeloid leukemia
CR: complete remission
DCL: donor cell‐derived leukemia
GVHD: graft‐versus‐host‐disease
STR: short tandem repeat
TAC: tacrolimus

## 1 INTRODUCTION

Donor cell‐derived leukemia (DCL), defined as the development of leukemia from donor cells after allogeneic hematopoietic stem cell transplantation (allo‐HSCT), is extremely rare [1, 2, 3, 4, 5]; limited evidence exists on the etiology of DCL, associated genetic aberrations, risk factors for its development, and prognosis. A standard treatment for DCL has not been established as yet. Herein, we report a case of *RUNX1*‐*RUNX1T1*‐positive DCL that developed after unrelated allogeneic bone marrow transplantation for DEK‐NUP214‐positive acute myeloid leukemia (AML).

## 2 CASE

**FIGURE 1:**
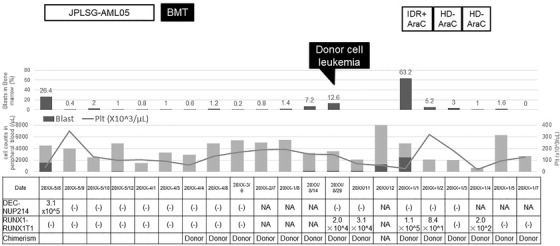
Clinical course of a patient with volunteer unrelated donor cell‐derived acute myeloid leukemia with RUNX1‐RUNX1T1. AML, acute myeloid leukemia; AraC, cytarabine; BMT, bone marrow transplantation; HD, high‐dose; IDR, idarubicin; JPLSG, Japanese Pediatric Leukemia/Lymphoma Study Group; ; NA, not available; Plt, platelet; (‐), not detected.

A 15‐year‐old male was diagnosed with AML with t(6;9)(p23;q34.1), a chimeric *DEK‐NUP214* fusion gene with an FLT3‐ITD mutation, in the pediatric department of our hospital in July 20XX‐5. From August 20XX‐5, systemic chemotherapy was started according to a pediatric protocol for AML (JPLSG‐AML05) and he subsequently achieved the first complete remission (CR). However, his leukemia was judged to be high risk for relapse, and the patient was referred to our department in order to receive allo‐HSCT. In March 20XX‐4, he underwent an allogeneic bone marrow transplantation (allo‐BMT) from a volunteer unrelated, fully matched (eight of eight loci) male donor at the first molecular CR. The conditioning regimen included intravenous busulfan (12.8 mg/kg total dose) and cyclophosphamide (120 mg/kg total dose). Prophylaxis for graft‐versus‐host disease (GVHD) included tacrolimus (TAC, 0.03 mg/kg initial dose) and short‐term methotrexate. He did not have any sign of acute GVHD, but experienced chronic mild GVHD of the skin that was controlled with topical steroids. TAC was discontinued on day 165 after allo‐BMT. Thereafter, the maintenance of molecular CR was confirmed by bone marrow specimens, using *DEK‐NUP214* chimeric gene testing (PCR), for 2 years following allo‐BMT. Of note, chimerism analysis using a short tandem repeat (STR) method on bone marrow specimens, carried out annually after allo‐BMT, indicated maintenance of the donor type (Figure 1).

During a routine examination in August 20XX (about 4.5 years after allo‐BMT), however, blasts were suddenly observed on a peripheral blood smear, with a white blood cell (WBC) count of 3200/μl (blasts, 5.0%), hemoglobin level of 14.2 g/dl, and platelet count of 152,000/μl, and a detailed examination was subsequently initiated accordingly (Table 1). Multiple bone marrow aspirations showed increased blasts of up to 63%, which were positive for myeloperoxidase, CD13, CD33, CD56, and CD34. Surprisingly, chromosome banding analysis showed 46, XY, t(8;21)(q22;q22.1) [12/20], and chimeric mRNA gene analysis showed the fusion of *RUNX1‐RUNX1T1* in bone marrow samples. However, *DEK‐NUP214* and *FLT3‐ITD*, which had been present at the time of the diagnosis of leukemia, were not detected. Finally, volunteer unrelated DCL was definitively diagnosed by multiple chimerism analysis using not only whole bone marrow samples, but also CD34‐positive cells which were selected by immunomagnetic beads.

**TABLE 1:** Laboratory findings at diagnosis in a patient with donor cell‐derived acute myeloid leukemia harboring a chimeric RUNX1‐RUNX1T1 fusion gene

Complete blood count		Biochemistry		Serology		
WBC	3200/μl	TP	6.8 g/dl	CRP	0.04 mg/dl	
Blast	5%	Alb	4.9 g/dl	IgG	639 mg/dl	
Neutro	44%	AST	13 U/L	IgA	112 mg/dl	
Mono	5%	ALT	12 U/L	IgM	62 mg/dl	
Lym	45%	LDH	126 U/L			
RBC	453 × 10⁴/μl	γ‐GTP	16 U/L	Bone marrow		
Hb	14.2 g/dl	ALP	186 U/L	Blast	7.2%	
Ht	41.8%	Cr	0.62 mg/dl	FCM	CD13+, CD33+, MPO+, HLA‐DR+	
MCV	92.2 fl	UA	5.0 mg/dl			
Ret	8‰	BUN	9.6 mg/dl	Karyotype	t(8;21)(q22;q22.1)	
Plt	15.2 × 10⁴/μl	Glc	97 mg/dl	RUNX1‐RUNX1T1		Detected
		Na	139 mmol/L	DEK‐NUP214		Not detected
Coagulation tests		K	4.0 mmol/L	FLT3‐ITD/TKD mutation		Not detected
PT‐INR	0.96	Cl	102 mmol/L			
APTT	106.6%	Ca	9.8 mg/dl	Chimerism	Whole	Donor type
Fib	275 mg/dl	T.Bil	1.2 mg/d		CD34 sorting	Donor type

Although the patient was diagnosed as having DCL, he was asymptomatic with a small number of blasts and did not wish to receive salvage chemotherapy (Figure 1). Therefore, he was followed up by bone marrow examination on an outpatient basis. The patient was admitted to our hospital with a cough, sputum, and fever in December 20XX. Blood tests showed a WBC count of 8300/μl (neutrophils, 62%; blasts, 15%) and a C‐reactive protein level of 19.4 mg/dl. Computed tomography revealed small diffuse infiltrates in both lungs, and infiltrates in the right inferior lobe as well as enhanced bronchial shadows. The patient was hospitalized for treatment of pneumonia. The pneumonia improved after antimicrobial therapy, but blasts increased in both peripheral blood and bone marrow. Therefore, remission induction therapy for DCL (idarubicin 12 mg/m^2^ on days 1–3 and cytarabine 100 mg/m^2^ on days 1–7) was started from January 11, 20XX+1. The patient developed bacteremia due to *Escherichia coli* during the period of bone marrow suppression, which improved with antimicrobial therapy. From February 21, 20XX+1, high‐dose cytarabine therapy (cytarabine 2 g/m^2^ twice daily on days 1–5) was started as a consolidation setting. Febrile neutropenia occurred, but improved after antibacterial and antifungal treatments. From March 26, 20XX+1, a second course of high‐dose cytarabine therapy was started at the same dosage as the first course. The patient developed febrile neutropenia, followed by severe pneumonia and hemophagocytic syndrome, with noninvasive positive‐pressure ventilation required in the intensive care unit. Consolidation therapy was terminated with the second course due to severe infection. The patient achieved and retained molecular CR on a *RUNX1–RUNX1T1* chimeric gene analysis in December 20XX+1.

## 3 DISCUSSION

The concept of DCL was first proposed in 1971 when Fialkow et al. reported a case that developed between relatives following allo‐BMT for acute lymphoblastic leukemia (ALL) [1]. Few reports exist on the incidence of DCL in large‐scale studies. However, of the 46,051 allogeneic transplants registered with the European Society of Blood and Marrow Transplantation (EBMT), only 38 were DCL cases (approximately 80.5 in 100,000 patients) [5]. In Japan, 40 out 36,870 allogeneic transplants were DCL cases (approximately 108.5 in 100,000 patients) according to the Japan Society for Hematopoietic Cell Transplantation [3]. Thus, although DCL is an extremely rare disease, its concept has become widely known. Nonetheless, no definitive evidence is available regarding the etiology, prognosis, and treatment of DCL due to the limited number of cases and abundance of background factors potentially involved in the pathogenesis.

DCL is diagnosed by G‐banding and cross‐sex fluorescence in situ hybridization for heterosexual transplants, but problems exist that are associated with age‐related Y chromosome defects and X chromosome polyploidy as an underlying condition. For homosexual transplants, chimerism can be analyzed by PCR amplification of STRs.

In our case, a definitive diagnosis of DCL was made approximately 5 years after allo‐BMT from an unrelated volunteer donor. The *DEK‐NUP214* chimeric gene and FLT3‐ITD mutation, which had been present when AML was first diagnosed, were not detected when DCL was diagnosed. Of note, the *RUNX1‐RUNX1T1* chimeric gene, which had not been present, was detected. Importantly, our patient underwent a periodic bone marrow examination on an annual basis after the allo‐BMT, with the *RUNX1‐RUNX1T1* chimeric gene, as well as the *DEK‐NUP214* chimeric gene, followed up for 2 years after allo‐BMT (Figure 1). Posttransplant chimerism STR analysis indicated maintenance of the donor type (Figure 1). Furthermore, chimerism STR analysis, performed on specimens obtained by sorting bone marrow cells with CD34, indicated the donor type (Table 1).

The clinical characteristics and outcome of the five patients including our case with DCL harboring *RUNX1‐RUNX1T1* are summarized in Table 2 [6, 7, 8, 9]. The disease occurred at a relatively young age in all five cases. The median time from transplantation to the onset of DCL was 34 months (range: 15–132 months). Only one patient underwent a second transplantation, and all patients showed DCL of a related donor origin except for this case. To the best of our knowledge, this is the first case of a volunteer unrelated donor cell‐derived AML harboring a chimeric *RUNX1‐RUNX1T1* fusion gene.

**TABLE 2:** Summary of five patients with donor cell‐derived acute myeloid leukemia harboring a chimeric RUNX1‐RUNX1T1 fusion gene

Case	Original disease	Age/sex (R)	Age/sex (D)	Donor sources	Conditioning	DCL type	Time from first HSCT to DCL (months)	Cytogenetic (G‐banding) analysis	Second HSCT	Outcome	Follow‐up period (months)	Ref.
1	AML	22/M	NA/F	rBM	CY	AML	34	45,X, t(8;21)	Yes	Alive, CR	17	[6]
2	CML	29/M	NA/F	rBM	CY	AML	132	45,X, t(8;21)	No	Death	1	[7]
3	ALL	24/F	NA/M	rBM	TBI, TLI, CY, ETP, MEL, AraC	AML	15	46,XY, t(8;21)	No	Death	NA	[8]
4	SAA	25/M	28/M	rPBSC	CY, ATG	AML	30	46,XY, t(8;21),+8	No	Alive, CR	36	[9]
5	AML	15/M	NA/M	uBM	BU+CY	AML	53	46,XY, t(8;21)	No	Alive, CR	12	This case

Abbreviations: ALL, acute lymphoblastic leukemia; AML, acute myeloid leukemia; AraC, cytarabine; ATG, anti‐thymocyte globulin; BU, busulfan; CML, chronic myeloid leukemia; CR, complete remission; CY, cyclophosphamide; D, donor; DCL, donor cell leukemia; ETP, etoposide; HSCT, hematopoietic stem cell transplant; MEL, melphalan; NA, not available; TLI, total lymphoid irradiation; Ref., reference; rBM, related bone marrow; R, recipient; rPBSC, related peripheral blood stem cell; SAA, severe aplastic anemia; TBI, total body irradiation; uBM, unrelated bone marrow.

The clinical characteristics and outcome of the seven patients including our case with DCL derived from unrelated volunteer donors are also summarized in Table 3 [8, 10, 11, 12, 13, 14]. The median ages of recipients were 34 years (range: 15–42). Bone marrow and peripheral blood were used as the donor source in four and two cases, respectively, while the donor source was unknown in the remaining one case. The median time from transplantation to the onset of DCL was 27 months (range: 5–193), with the second transplantation being carried out in only one of five cases for which the relevant information was available. With a median follow‐up of 10 months (range: 1–30), two of the seven patients died.

**TABLE 3:** Summary of seven patients with unrelated volunteer donor cell‐derived acute myeloid leukemia

Case	Original disease	Age/sex (R)	Age/sex (D)	Donor source	Conditioning	DCL type	Time from first HSCT to DCL (months)	Cytogenetic (G‐banding) analysis	Second HSCT	Outcome	Follow‐up period (months)	Ref.
1	CML‐CP	35/M	NA/M	uBM	BU, CY, TT	AML	39	46,XY t(15;17)	No	Alive	30	[8]
2	CML‐CP	40/M	NA/M	uBM	BU, CY, ATG	AML	14	46,XY	Yes	Alive	9	[10]
3	M. Sar	34/F	37/M	uBM	CY, TBI, ALZ	AML	27	45,XY,‐7	No	Dead	5	[11]
4	CML‐CP	42/M	NA/F	uPB	MA	AML	193	46,XX	No	Alive	1	[12]
5	NA	20/M	NA/M	u‐NA	CY, TBI	AML	5	NA	NA	NA	NA	[13]
6	ALL	28/F	33/M	uPB	BU, CY, AraC, Semustine, ATG	AML	20	46,XY	NA	Dead	11	[14]
7	AML	15/M	NA/M	uBM	BU+CY	AML	53	46,XY, t(8;21)	No	Alive, CR	12	This case

Abbreviations: ALL, acute lymphoblastic leukemia; ALZ, alemtuzumab; AML, acute myeloid leukemia; AraC, cytarabine; ATG, anti‐thymocyte globulin; BU, buslfan; CML‐CP, chronic myeloid leukemia, chronic phase; CR, complete remission; CY, cyclophosphamide; D, donor; DCL, donor cell leukemia; F, female; HSCT, hematopoietic stem cell transplantation; M, male; MA, myeloablative; M. Sar, myeloid sarcoma; NA, not available; R, recipient; Ref., reference; uBM, unrelated bone marrow; TBI, total body irradiation; TT, thiotepa; uPB, unrelated peripheral blood stem cell.

According to an EBMT report, the prognosis of DCL is poor, with 29 of 38 patients dying at a median of 11 months (range: 0–91 months) following a DCL diagnosis [5]. Of the 38 patients, 18 underwent a second transplantation. The cause of death was DCL in 12 patients, while 10 cases were transplantation‐related deaths. The high incidence of death from DCL progression warrants the consideration of allo‐HSCT, but a consensus on this has not yet been reached. Our patient developed AML harboring a chimeric *RUNX1‐RUNX1T1* fusion gene approximately 5 years after allo‐BMT. In general, RUNX1‐RUNX1T1‐positive AML is a core‐binding factor AML for which high‐dose cytarabine is effective as consolidation therapy [15], but the usefulness of allo‐HSCT in first CR has not been demonstrated. Accordingly, high‐dose cytarabine therapy was given after induction therapy, and a second transplantation was not performed in the present case. Nevertheless, since the prognosis of DCL has been shown to be relatively poor, as described above, it is necessary to further accumulate clinical characteristics and outcome regarding DCL to make treatment decisions appropriately.

## CONFLICT OF INTEREST

Shinsuke Iida received grants and personal fees from Celgene, grants and personal fees from Janssen, grants and personal fees from Ono, grants and personal fees from Takeda, grants and personal fees from Bristol‐Myers Squibb, grants from Chugai, grants from Kyowa Kirin, grants and personal fees from Sanofi, grants from Abbvie, grants and personal fees from Daiichi Sankyo, outside the submitted work.
